# Transcriptomic and metabolomic reveal OsCOI2 as the jasmonate-receptor master switch in rice root

**DOI:** 10.1371/journal.pone.0311136

**Published:** 2024-10-28

**Authors:** Mohamad Cheaib, Hieu Trang Nguyen, Marie Couderc, Julien Serret, Alexandre Soriano, Pierre Larmande, Chris Richter, Björn H. Junker, Manish L. Raorane, Anne-Sophie Petitot, Antony Champion

**Affiliations:** 1 DIADE, IRD, University Montpellier, Montpellier, France; 2 UMR AGAP Institut, CIRAD, INRAE, Institut Agro, University Montpellier, Montpellier, France; 3 Institute of Pharmacy, Martin-Luther-University, Halle-Wittenberg, Halle, Germany; Hainan University, CHINA

## Abstract

Jasmonate is an essential phytohormone involved in plant development and stress responses. Its perception occurs through the CORONATINE INSENSITIVE (COI) nuclear receptor allowing to target the Jasmonate-ZIM domain (JAZ) repressors for degradation by the 26S proteasome. Consequently, repressed transcription factors are released and expression of jasmonate responsive genes is induced. In rice, three *OsCOI* genes have been identified, *OsCOI1a* and the closely related *OsCOI1b* homolog, and *OsCOI2*. While the roles of OsCOI1a and OsCOI1b in plant defense and leaf senescence are well-established, the significance of OsCOI2 in plant development and jasmonate signaling has only emerged recently. To unravel the role of OsCOI2 in regulating jasmonate signaling, we examined the transcriptomic and metabolomic responses of jasmonate-treated rice lines mutated in both the *OsCOI1a* and *OsCOI1b* genes or *OsCOI2*. RNA-seq data highlight OsCOI2 as the primary driver of the extensive transcriptional reprogramming observed after a jasmonate challenge in rice roots. A series of transcription factors exhibiting an OsCOI2-dependent expression were identified, including those involved in root development or stress responses. OsCOI2-dependent expression was also observed for genes involved in specific processes or pathways such as cell-growth and secondary metabolite biosynthesis (phenylpropanoids and diterpene phytoalexins). Although functional redundancy exists between OsCOI1a/b and OsCOI2 in regulating some genes, *oscoi2* plants generally exhibit a weaker response compared to *oscoi1ab* plants. Metabolic data revealed a shift from the primary metabolism to the secondary metabolism primarily governed by OsCOI2. Additionally, differential accumulation of oryzalexins was also observed in *oscoi1ab and oscoi2* lines. These findings underscore the pivotal role of OsCOI2 in jasmonate signaling and suggest its involvement in the control of the growth-defense trade-off in rice.

## Introduction

Jasmonate (JA) is an essential phytohormone involved in the regulation of plant growth and development as well as in responses to biotic and abiotic stress [1–3]. JA perception occurs mainly through its bioactive form jasmonoyl-isoleucine (JA-Ile), although other JA-amino acids conjugates can function as endogenous JA bioactive molecules [4, 5]. In Arabidopsis, JA perception is mediated by a unique nuclear receptor CORONATINE INSENSITIVE1 (COI1), which is an F-box protein component of the Skp1-Cul-F-box protein (SCF)-type E3 ubiquitin ligase complex or SCF^COI1^ [6–8]. Upon JA perception, COI1 interacts with nuclear Jasmonate-ZIM domain (JAZ) repressors, allowing the SCF^COI1^ complex to target the JAZs for ubiquitination and degradation by the 26S proteasome. Elimination of JAZ proteins releases the JAZ-repressed transcription factors (TFs), such as MYC2, and promotes the expression of JA-responsive genes [9–11]. Modularity in the core JA signaling pathway, that is, the combinations of JA-derivative ligands, JAZs and TFs, allows for the integration of multiple input signals to mediate various and specific transcriptional responses adapted to specific demand [12].

COI1, as a JA receptor, is a critical protein for signal transduction. In contrast to dicotyledonous plants, the COI receptor is encoded by a small gene family in the genomes of monocotyledonous plants. In maize, four *COI* genes were initially identified [13] followed by the description of six *COI* genes, among them four COI1 genes, named *ZmCOI1a*, *ZmCOI1b*, *ZmCOI1c* and *ZmCOI1d*, and two COI2 genes, named *ZmCOI2a* and *ZmCOI2b* [14]. In rice, three *COI* genes have been identified, *OsCOI1a* and the closely related *OsCOI1b* homolog, and *OsCOI2* [15]. All the *OsCOI1* and *ZmCOI1* genes are able to complement the Arabidopsis *coi1-1* mutant to restore fertility and JA signal transduction, suggesting they are the functional orthologs of COI1 in rice and maize, respectively [13, 15]. In rice, a role for *OsCOI1a* and *OsCOI1b* genes has been shown in plant defense, for instance in antiviral defense through the control of the RNA silencing protein AGO18 [16], in the control of basal resistance during infection by the rice blast fungus *Magnaporthae oryzae* [17], or in anti-herbivore defense when rice is attacked by the rice leaf-folder insect [18]. A role for *OsCOI1b* was also associated to leaf senescence [19].

As *OsCOI2* and *ZmCOI2* genes were not able to complement the *coi1-1* mutation in Arabidopsis, they were predicted to have divergent functions. However, the characterization of *oscoi2* and *zmcoi2* edited lines revealed their role in plant development and JA signaling. *ZmCOI2a* and *ZmCOI2b* were notably shown to redundantly regulate anther dehiscence, pollen development and male fertility [14]. In rice, three recent and independent studies converged to demonstrate the major role for *OsCOI2* in plant development and JA signaling [20–22]. A crucial role for OsCOI2 in rice fertility was demonstrated and characterized in *oscoi2* mutants by a default in anther dehiscence and a low pollen germination rate [20–22]. OsCOI2 was shown to regulate different JA responses: (i) root growth inhibition by JA was abolished in *oscoi2* mutants [20–22] and (ii) JA-responsive marker genes and defense genes were expressed in an *OsCOI2*-dependent manner [20, 22]. Furthermore, an important role for OsCOI2 was also identified in defense mechanisms against bio-aggressors. Enhanced sensitivity to the brown planthopper (BPH) insect, a leaf rice pest, was observed in the *oscoi2* mutant and accumulation of some antimicrobial secondary metabolites, either phytoalexins or phenylpropanoids, was suppressed in the *oscoi2* mutants in response to JA or to BPH attack, respectively [20, 21].

Our main objective was to decipher the molecular responses controlled by the OsCOI receptors in rice roots, where differential root length inhibition and defense responses occurred in response to JA in the edited *oscoi* lines compare to WT plants [22]. Here, combined transcriptome, metabolome and genetic analyses were used to study the JA-dependent root responses regulated by OsCOI1a/b and OsCOI2, and to identify the major roles played by OsCOI2 in JA-dependent developmental and defense responses.

## Results

### Whole genome sequencing of the *oscoi* edited lines

Before performing transcriptomic analyses, we evaluated the potential off-target effects of CRISPR/Cas9 in the *oscoi1ab* (both edited in the *OsCOI1a* and *OsCOI1b* genes) and *oscoi2* lines. We carried out whole genome sequencing on both lines as well as on the Kitaake wild-type (WT) line we used for transformation and genome editing. First, we performed global alignments of the chromosomes sequences to verify that there was no rearrangement due to the edition approach or to the regeneration process (S1 Fig and S1 Table). Then, we looked for potential off-target sites to verify if any mutation occurred at these locations. We confirmed edition of the targeted genes and mutations previously described in *oscoi1ab* and *oscoi2* lines (S2 Table) [22]. While nucleotide insertions were detected at a few off-target sites, no protein-encoding genes was affected (S2 Table).

### Transcriptomes of the *oscoi* edited lines

We performed RNA-seq analyses of crown root tips from WT plants, *oscoi1ab* and *oscoi2* mutant lines treated or not by JA. Between ~33 and 37 M read pairs were obtained for each library and mapped on the *Oryza sativa* reference genomes (S3 Table). A global comparison using principal component analysis revealed that the double mutant line *oscoi1a/b* exhibits a transcriptome pattern close to that of the WT, which is distinguishable from the transcriptome of the *oscoi2* mutant line under both mock and JA treated conditions (S2 Fig).

### Differential gene expression between *oscoi* lines and WT plants under mock condition

**Fig 1:**
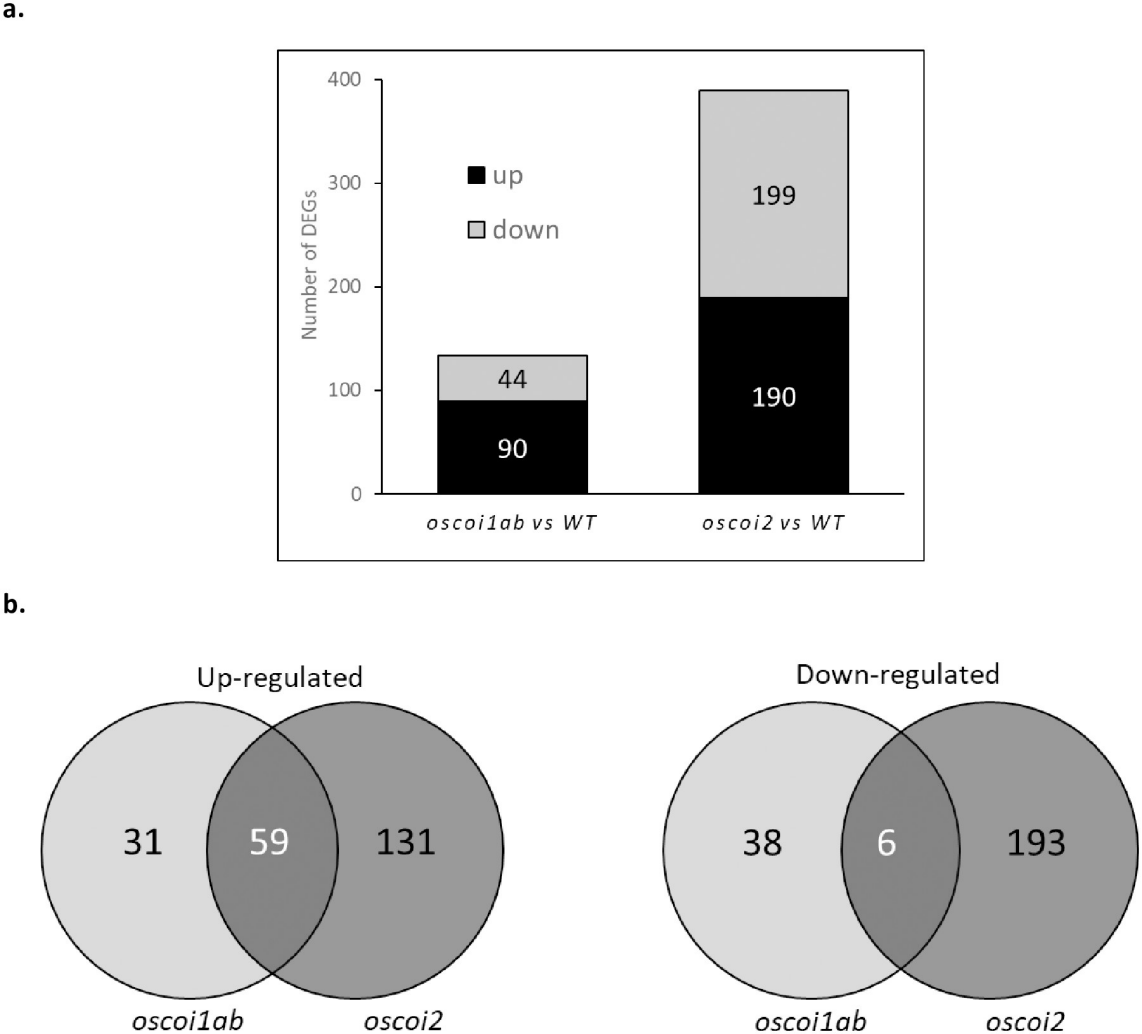
Number of DEGs between *oscoi* lines and WT plants. **a.** Number of DEGs identified in crown root tips from *oscoi1ab* and *oscoi2* lines compared to WT plants. **b.** Venn diagrams showing specific and common DEGs found in *oscoi1ab* and *oscoi2* lines compared to WT plants.

To identify any OsCOI regulated genes in absence of JA treatment, we first looked for differentially expressed genes (DEGs) occurring between untreated plants (Fig 1a and S4 Table). Between *oscoi1ab* and WT crown root tips, 134 DEGs were identified. Among the 90 up-regulated genes, we notably identified *OsJAZ15* and one gene encoding a TF, *OsERF96*. Among the 44 down-regulated genes, we identified *OsCOI1a*, what can be correlated to the edition event, and one gene encoding for a TF, *OsTCP25*. Between *oscoi2* and WT crown root tips, more differences were observed with a total of 389 DEGs. Among the 190 up-regulated genes, we notably identified 4 genes encoding dirigent proteins and 2 genes encoding TFs (*OsbHLH186* and *OsERF96*). Among the 199 down-regulated genes, we identified a high number of genes encoding TFs, i.e. 16 genes (listed in S4 Table), suggesting that OsCOI2 is a direct or indirect positive regulator of these TFs. Venn diagrams showed that few genes are common DEGs between *oscoi1ab* and *oscoi2* when compared to WT plants (Fig 1b). Among the common up-regulated genes, we specially identified 5 NB-ARC genes and one pectin lyase (*OsPME1*). Among the common down-regulated genes, 2 genes encoding lipid-transfer proteins (*OsLTP2* and *OsLTPIV*) and 2 genes encoding TFs (*OsERF96* and *OsTCP25*) were identified. These data suggest that these genes may be co-regulated by OsCOIs receptors in rice root tips.

### Gene expression profiling of *oscoi* lines subjected to JA treatment

**Fig 2:**
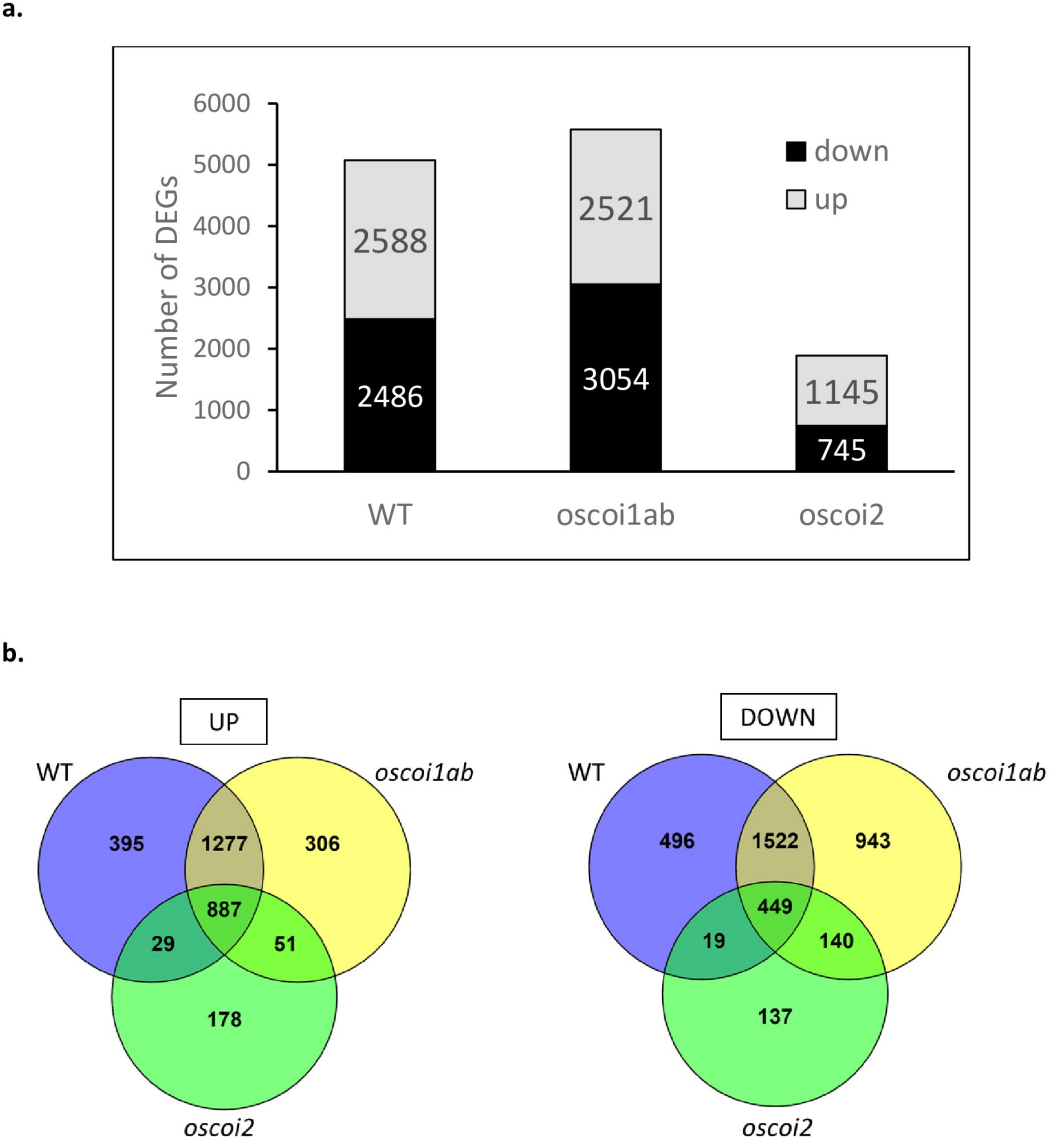
Number of DEGs in response to JA treatment in WT plants and *oscoi* lines. **a.** Number of DEGs in crown root tips after 6h JA treatment (5 μM). **b.** Venn diagrams showing specific and common DEGs in WT plants and *oscoi* lines in response to JA. Up: up-regulated genes, down: down-regulated genes.

The JA effect on gene expression in crown root tips of the WT plants, was analyzed. A total of 5074 DEGs was identified, including 2588 up-regulated genes and 2486 down-regulated genes (Fig 2a and S5 Table). For the *oscoi1ab* plants treated by JA, a similar number of DEGs were recorded with a total of 5575 DEGs, including 2521 up-regulated genes and 3054 down-regulated genes. The *oscoi2* plants showed less differences in gene expression under JA treatment, as only 1890 DEGs were identified, including 1145 up-regulated genes and 745 down-regulated genes.

WT plants shared the highest number of DEGs with the *oscoi1ab* plants, i.e. 84% (1277+887 DEGs) of the up-regulated genes and 79% (1522+449 DEGs) of the down-regulated genes, whereas they shared only 35% (29+887 DEGs) of the up-regulated genes and 19% (19+449 DEGs) of the down-regulated genes with the *oscoi2* plants (Fig 2b). In terms of specificity, 49% (1277 DEGs) and 61% (1522 DEGs) of the genes defined as up- and down-regulated genes respectively, in both WT and *oscoi1ab* plants were not deregulated in *oscoi2* plants, indicating an *OsCOI2*-dependent expression after JA treatment. In contrast, only 1% of the DEGs (29 up-DEGs and 19 down-DEGs) displayed an *oscoi1ab*-dependent expression. Either functional redundancy of the OsCOI receptors or OsCOI-independent pathways can be related to the common DEGs, accounting for 34% (887 DEGs) of the up-regulated genes and 18% (449 DEGs) of the down-regulated genes. Finally, 15% (395 up-DEGs) or 20% (496 down-DEGs) of the DEGs were only detected in the WT plants upon JA treatment, suggesting a gene regulation that requires the three *OsCOI* genes. As a main result, we retained that the majority of transcriptional changes upon JA treatment in rice roots is OsCOI2-dependent. RT-qPCR experiments performed on 11 genes validated the RNA-seq data (S3 Fig).

### Expression of genes related to JA metabolism and perception

**Fig 3:**
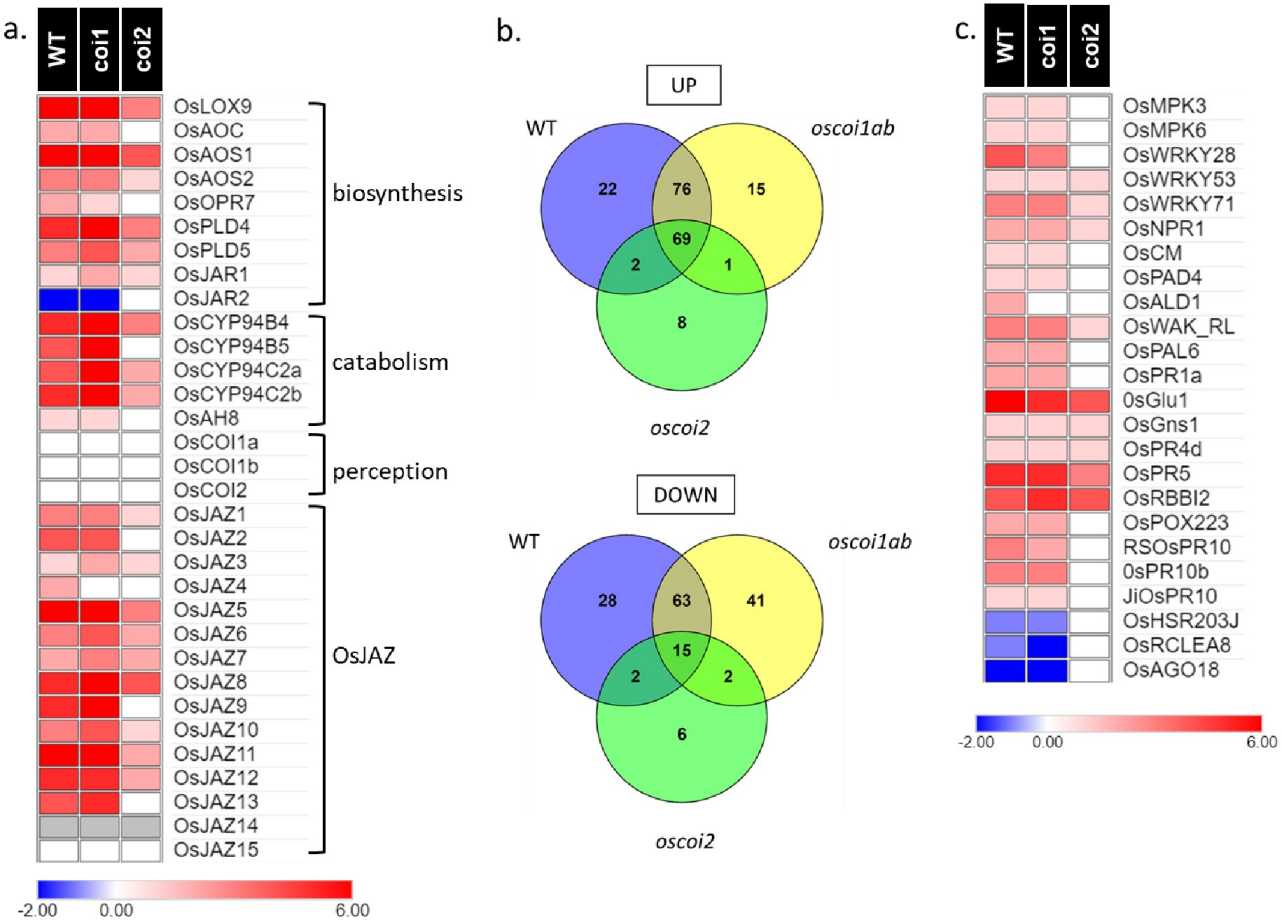
Expression of JA-related genes in response to JA treatment in WT plants and in *oscoi* mutants. **a.** Expression pattern of genes related to JA metabolism and perception **b.** Venn diagrams showing specific and common DEGs encoding for transcription factors (Up: up-regulated genes, down: down-regulated genes). **c.** Expression pattern of genes related to plant defense. For heatmaps, the key color represents the log_2_FC values of the DEGs identified between JA-treated samples and controls samples (see S6 Table for detailed values).

On the 14 genes associated to JA metabolism (biosynthesis and catabolism), we found that 13 were induced by JA treatment in WT plants while only one, *OsJAR2*, is repressed (Fig 3a and S6 Table). These genes displayed the same expression patterns in *oscoi1ab* plants. In *oscoi2* plants, upon JA treatment most of these genes exhibited a weaker response with smaller log_2_FC values than the WT and *oscoi1ab* plants. Five of them behaved similar than in the mock treated plants indicating that these genes are OsCOI2-dependent genes.

Concerning the expression of genes involved in JA perception, we observed that the *OsCOI* genes are not induced by JA treatment in crown root tips of any plant lines as shown in our previous data (Fig 3a and S6 Table) [22]. Among the *OsJAZ* genes, *OsJAZ14* is not detected in crown root tips and *OsJAZ15* is only detected in the *oscoi1ab* mutant. All the other *OsJAZ* genes are significantly induced by JA treatment in WT plants (log_2_FC from ~2 to 6.8) or in the *oscoi1ab* mutants, except *OsJAZ4*, with similar induction levels (log_2_FC from 2.5 to 8.7). In the *oscoi2* mutant, most of the *OsJAZs* are induced by JA treatment but at weaker levels than in WT plants (log_2_FC from 1.3 to 4.2). Importantly, *OsJAZ-2*, *-9* and -*13* show no significant induction by JA treatment in the *oscoi2* line, revealing an OsCOI2-dependent expression. *OsJAZ4* is not induced in the *oscoi1ab* and *oscoi2* lines revealing an OsCOI1- and OsCOI2-dependent expression.

### OsCOI-dependent transcription factors modulated by jasmonate in roots

To estimate the transcriptional changes regulated by OsCOI1a, OsCOI1b and OsCOI2, we investigated the regulation of TFs expression in *oscoi1ab* and *oscoi2 lines*. First, we mapped all the DEGs to the PlantTFDB79 (http://planttfdb.gao-lab.org/) to retrieve JA associated TFs. This analysis revealed that, on the 1362 genes annotated as TFs in *O*. *sativa*, there are 277 DEGs (20%) in WT plants upon JA treatment, 283 DEGs (21%) in *oscoi1ab* plants and only 105 DEGs (8%) in *oscoi2* plants (S7 Table).

Fifty TFs (28 TFs up and 22 TFs down) are regulated by JA in WT roots and not in either *oscoi1ab* or *oscoi2* mutant lines suggesting that all three receptors are necessary to control expression of these genes (Fig 3b). In addition, 84 TFs are regulated in a similar manner (69 TFs up et 15 TFs down) both in WT and *oscoi* mutants but as observed for *OsJAZ* genes with a different expression in *oscoi2* mutant in response to JA (Fig 3b). For example, TFs playing a well-characterised role in JA signalling, such as *OsMYC2* and *OsJAMYB*, are induced in WT and *oscoi* mutant plants, but with a significant lower level of expression in *oscoi2* mutant suggesting partial redundancy among OsCOI receptors (S7 Table).

Remarkably, fifty percent of TFs (139 TFs) were specifically regulated in an OsCOI2-dependent manner (i.e. differentially expressed in WT and *oscoi1ab* mutant but not in *oscoi2* mutant), while only 4 TFs were regulated via OsCOI1s receptors. To identify the biological functions associated with each TF regulated by OsCOI receptors, we performed an in-depth analysis of published data related to the TF genes mostly based on the effect of mutation (loss of function) or overexpression on plant development and/or response to stresses (S7 Table). Several TFs induced by JA and strictly controlled by OsCOI2 are known to play a role in root development such as *OsERF40*, *OsERF2*, *OsNAC39* and *OsWOX6*. Others have been described to be involved in responses to abiotic stresses such as drought (*OsbHLH148*, *OsADR3* and *OsONAC79*), cold (*OsMYB4* and *OsOBF1*) or salt stress (*OsMYB106* and *OsNAC45*) and responses to pathogens (*OsMYB30*, *OsMYB110* and *OsDPF*). Three TFs (*OsbHLH148*, *OsMYB30* and *OsSPL2*) are also known to interact with the OsJAZ repressors and thus could participate to OsCOI2 JA-dependent signaling pathways. Among the TFs repressed by JA treatment in roots, several are known to play a role in development, photosynthesis and in response to abiotic stresses (S7 Table). Among the four OsCOI1-dependent TFs in response to JA in the roots, we found *OsNAC2* and *OsHsfA7* involved in root growth and abiotic response (S7 Table).

Taken together, these results highlight the significant contribution of the OsCOI2 receptor in rewiring the transcriptional program of rice roots in response to JA stimulation.

### Expression of defense marker genes

Expression of a series of genes related to biotic stresses, involved either in signaling (kinases, TFs) or defense responses (PR proteins) was studied upon JA treatment. Among those, we identified 21 genes induced in crown root tips upon JA treatment, in WT plants as well as in *oscoi1ab* plants (Fig 3c and S6 Table). Only 9 of these genes were significantly induced in *oscoi2* plants, typically with a lower log_2_FC value than for WT and *oscoi1ab* plants. Among the *oscoi2*-dependent genes, we identified for instance, the MAP kinases *OsMAPK3* and *OsMAPK6* and all the studied PR10 genes. Three genes (*Oshsr203J*, *OsRCLEA8* and *OsAGO18*) that are repressed in WT and *oscoi1ab* plants are also *oscoi2*-dependent. Thus, expression analysis of these marker genes reveals a major role for OsCOI2, although functional redundancy happens for some genes.

### Metabolic pathways controlled by OsCOI2

**Fig 4:**
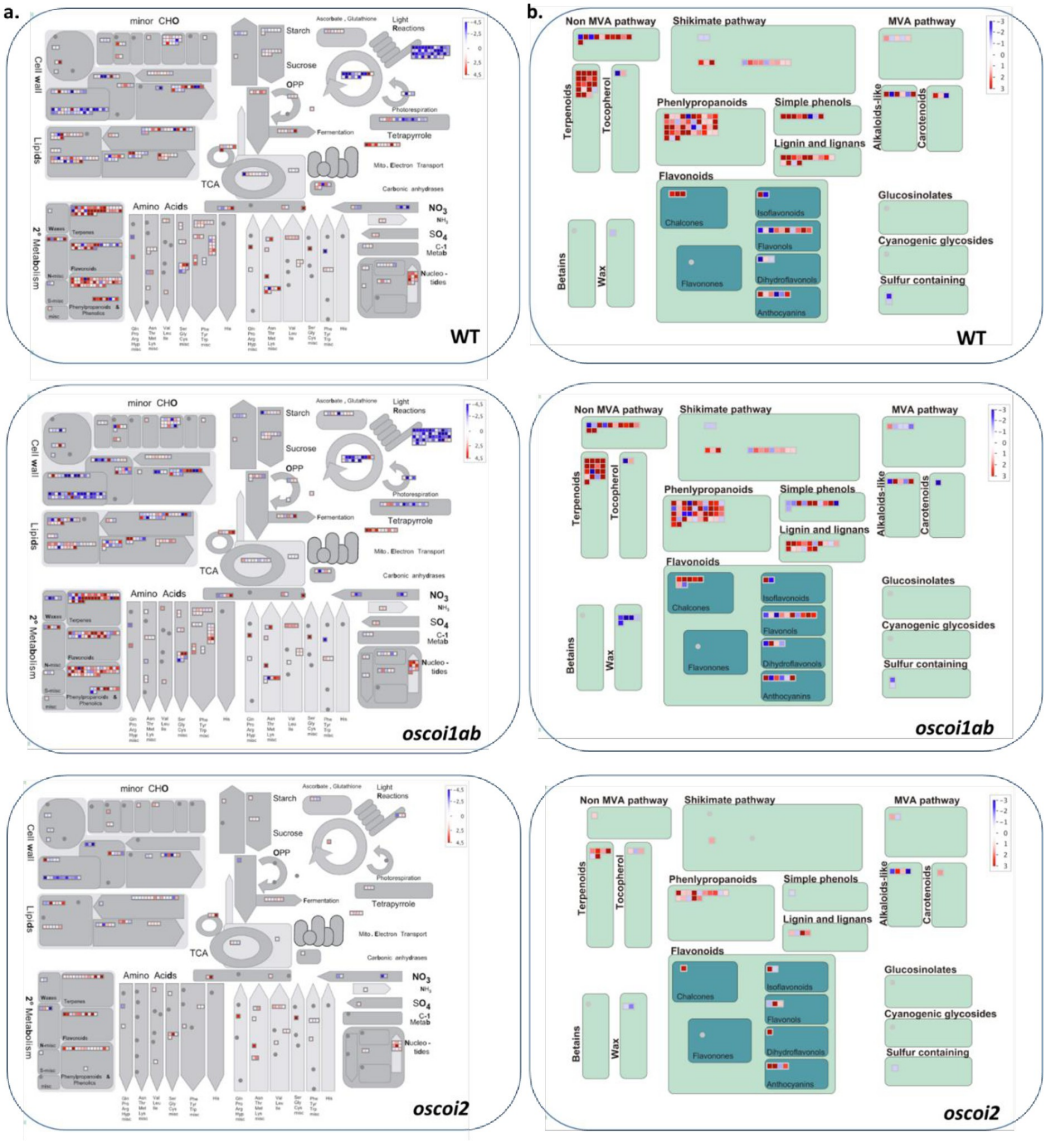
Mapman analysis of the DEGs identified in WT plants and *oscoi* lines in response to JA. **a.** Mapman metabolism overview. **b.** Mapman representation of genes associated to secondary metabolism pathways. The key color represents the log_2_FC values between JA-treated samples and controls samples.

To look for specific metabolic pathways controlled by OsCOI receptors under JA treatment, we performed Mapman analyses and Gene Ontology enrichments (Fig 4 and S8 Table). These analyses pointed to pathways displaying common expression patterns between WT and *oscoi1ab* plants whereas they were not deregulated or partially deregulated in *oscoi2* plants. Regarding the up-regulated genes, we found several pathways linked to secondary metabolic production and regarding the down-regulated genes, we identified pathways related to photosynthesis and to cell wall organization, as described above.

**Fig 5:**
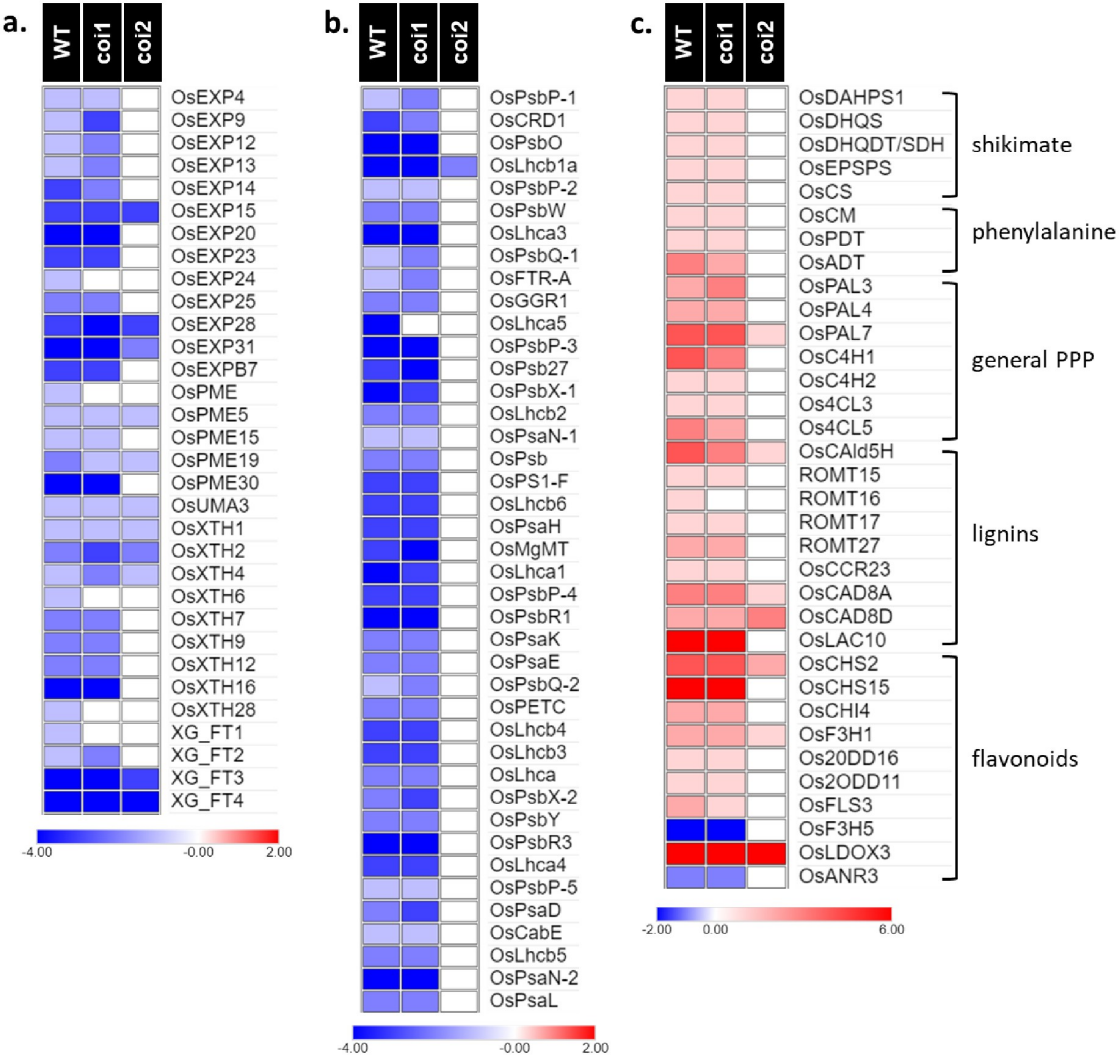
Metabolic pathways controlled by OsCOI2 in response to JA treatment. **a.** Expression pattern of genes related to cell expansion. **b.** Expression pattern of genes related to photosynthesis. **c.** Expression pattern of genes related to the phenylpropanoid pathway (PPP), including the shikimate pathway and the synthesis of phenylalanine, the PPP precursor. The key color represents the log_2_FC values of the DEGs identified between JA-treated samples and controls samples (see S6 Table for detailed values).

In the process “cell-wall organization or biogenesis”, we identified 32 down-regulated genes in WT plants in response to JA treatment (Fig 5a and S6 Table). These genes encode enzymes related to cell expansion or cell wall remodeling such as expansins (*OsEXP*), xyloglucan endotransglucosylase/hydrolases (*OsXTH*), pectin methylesterases (*OsPME*), UDP-arabinopyranose mutase (*OsUMA*) and xyloglucan fucosyltransferases (*XG_FT*). Most of these genes are also repressed in *oscoi1ab* plants while only 11 of these genes were detected as DEGs in *oscoi2* plants. The expression patterns of these genes can be linked to the root growth phenotypes, i.e. a ~60% growth reduction measured for WT and *oscoi1ab* plants upon JA treatment and a ~30% growth reduction observed for *oscoi2* plants [22].

The photosynthesis process was severely affected by JA treatment with 41 genes belonging to this category identified as down-regulated in WT plants (Fig 5b and S6 Table). Genes encoding Photosystem I and Photosystem II subunits (*OsPSa* and *OsPsb* genes) and genes encoding proteins from the light- harvesting complex (*OsLhca* and *OsLhcb* genes) were identified in this category. Forty genes were also repressed in the *oscoi1ab* plants but only one of these genes was repressed in the *oscoi2* plants. These data indicate that the repression of this major primary metabolic pathway in response to JA is OsCOI2-dependent.

Conversely, numerous genes related to secondary metabolites were induced by JA treatment. Genes involved in the biosynthesis of phenylpropanoids, such as lignin precursors and flavonoids, were induced in WT and *oscoi1ab* plants but not in *oscoi2* plants (Fig 5c and S6 Table). Genes from the general phenylpropanoid pathway (PPP) (*OsPAL*, *OsC4H* and *Os4CL*) as well as genes from the shikimate pathway leading to phenylalanine, the PPP precursor, display such expression pattern. *OsPAL7* represents an exception with a slight induction observed in *oscoi2* plants. Some genes involved in lignins *(OsROMT*, *OsCCR23* and *OsLAC10*) and flavonoids (*OsCHS15*, *OsCHI4*, *Os2ODD* and *OsFLS3*) biosynthesis are only induced in WT and *oscoi1ab* plants whereas other genes (*OsCald5H*, *OsCAD8*, *OsCHS2*, *OsF3H1* and *OsLDOX3*) are also induced in *oscoi2* plants. Finally, two genes (*OsF3H5* and *OsANR3*) were repressed only in WT and *oscoi1* plants. The *N-OMT* gene, encoding an N-O-methyl transferase leading to sakuranetin, the only rice phytoalexin from the flavonoid pathway, was not expressed in our dataset.

**Fig 6:**
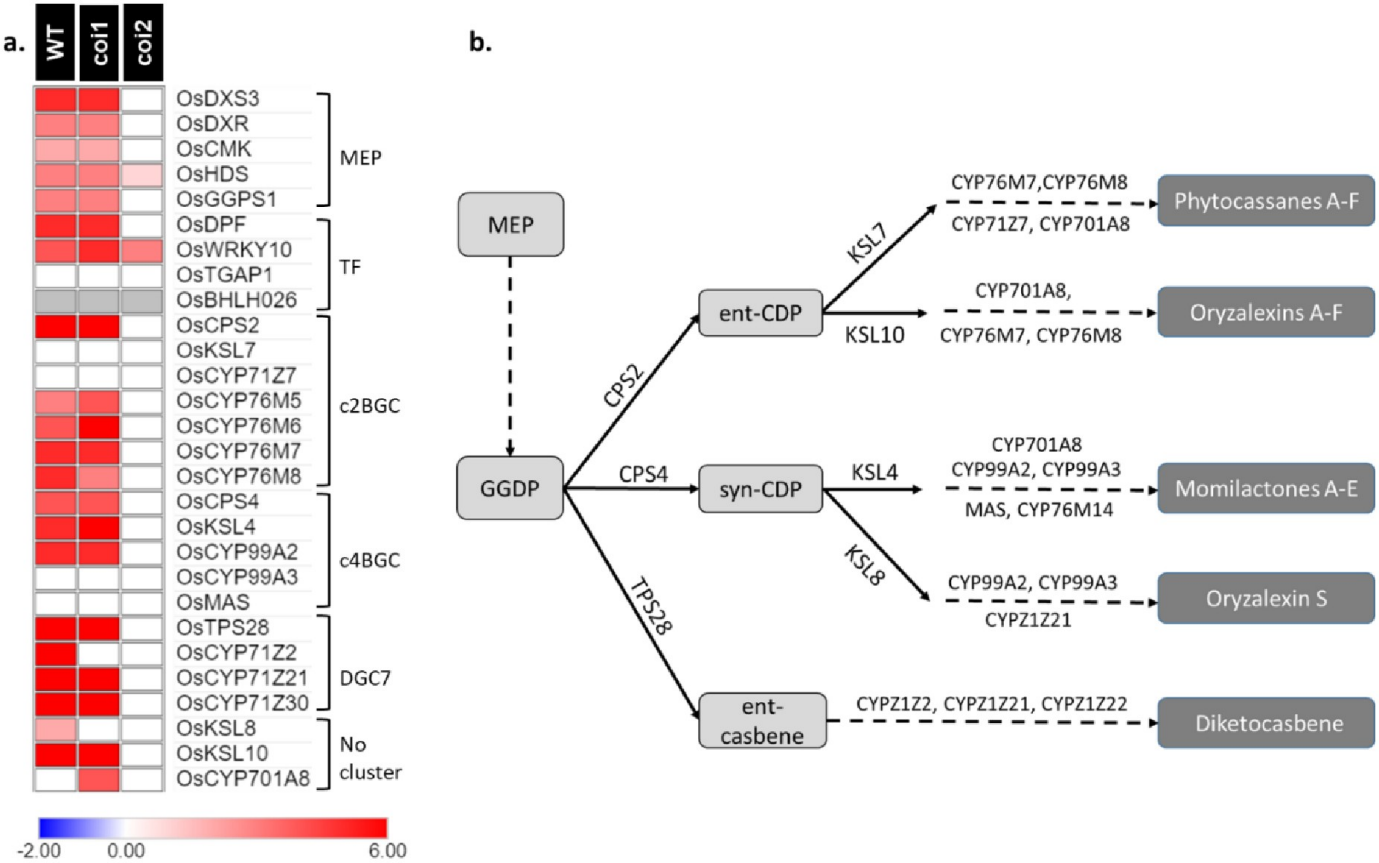
*OsCOI2*-dependent expression of Diterpene Phytoalexins (DP) biosynthesis genes. **a.** Expression pattern of genes related to the methylerythritol phosphate pathway (MEP), the known TFs involved in DP genes regulation, and DP biosynthesis genes located on the c2BGC (chromosome 2 biosynthesis gene cluster), on the c4BGC (chromosome 4 biosynthesis gene cluster) and on DGC7: (diterpenoid gene cluster on chromosome 7). The key color represents the log_2_FC values of DEGs between JA-treated samples and controls samples (see S6 Table for detailed values). **c.** Schematic representation of DP biosynthesis. Oryzalexin S biosynthesis was recently elucidated [23].

Besides genes from PPP, genes involved in diterpene phytoalexins (DP) biosynthesis, as well as genes from the methylerythritol phosphate pathway (MEP), leading to the phytoalexin precursor geranylgeranyldiphosphate (GGDP), were induced by JA treatment (Fig 6 and S6 Table). Four genes from the MEP pathway (*OsDXS3*, *OsDXR*, *OsCMK* and *OsGPPS1*) are up-regulated in WT and *oscoi1ab* plants but not in *oscoi2* plants while *OsHDS* is also induced in *oscoi2* plants but with a lower fold-change than in WT and *oscoi1ab* plants. Genes involved in the first steps of phytoalexins biosynthesis (*OsCPS2* and *OsCPS4*) and genes leading to oryzalexins (*OsKSL8* and *OsKSL10*) and momilactones (*OsKSL4*) as well as *OsCYP* genes involved in the latter steps of phytoalexins synthesis are also up-regulated in WT and *oscoi1ab* plants but not in *oscoi2* plants. Genes from the DGC7 cluster (*OsTPS28* and *OsCYP*) leading to diketo-casbene also displayed higher expression levels in WT and *oscoi1ab* plants. Among the four TFs recognized as regulators of DP biosynthesis, we found *OsDPF* as the sole regulator exhibiting JA-inducible expression dependent on OsCOI2. *OsWRKY10* demonstrated partial OsCOI2 dependence, with a more significant alteration observed in WT and *oscoi1ab* plants compared to *oscoi2* plants. However, the remaining two regulators, *OsTGAP1* and *OsbHLH026*, either showed no reaction to JA treatment or were undetectable in the root.

### Primary metabolism profiling of *oscoi* lines submitted to JA treatment

**Fig 7:**
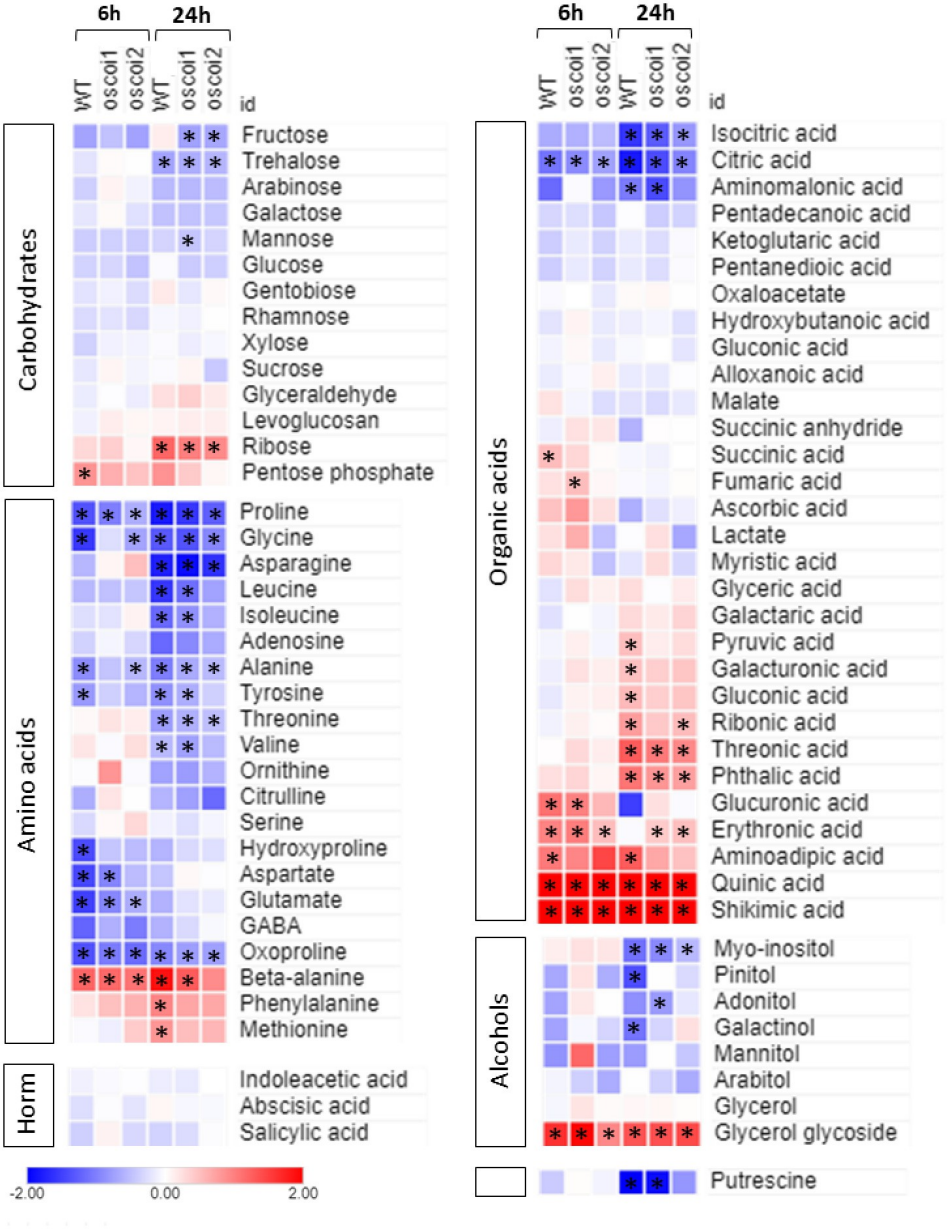
Metabolite accumulation in rice roots submitted to JA treatment. The key color represents the Log_2_FC values between JA-treated samples and controls samples. Stars indicate significant difference levels between JA-treated samples and controls samples (p < 0.01) (see S9 Table for detailed values).

The accumulation of various primary metabolites in rice roots treated with JA (6h and 24h) was measured by GC-MS: a total of 77 metabolites, including proteinogenic and non-proteinogenic amino acids, carbohydrates, organic acids, alcohols and some hormones, was detected and quantified (Fig 7 and S9 Table). Overall, WT plants exhibited a more pronounced change in metabolite accumulation after a 24-hour treatment (32 metabolites, approximately 42%) than after a 6-hour treatment (18 metabolites, approximately 23%).

The most important differences were found for amino acids. In WT plants, levels of most of them significantly decrease following a JA treatment, as soon as 6h for proline, glycine, alanine, tyrosine, hydroxyproline, aspartate, glutamate and oxoproline, or at 24h for asparagine, leucine, isoleucine, threonine and valine. On the contrary, the levels of beta-alanine, phenylalanine and methionine significantly increase after JA treatment. In the organic acid category, we observed significant reduction levels of citric acid, isocitric acid and amino-malonic acid, while a series of acids accumulates after JA treatment either at 6h (glucuronic, erythronic and aminoadipic acids) or 24h (pyruvic, galacturonic, gluconic, ribonic, threonic and phthalic acids). Remarkably, quinic acid and shikimic acid show the highest accumulation levels after JA treatment (Fig 7 and S9 Table). In the alcohols, myo-inositol, pinitol and galactinol levels decrease at 24h while glycerol glycoside levels increased from 6h to 24h. Among the sugars, few modifications occur: the trehalose level decreases after JA treatment while the pentose phosphate and the ribose levels increase. No modifications were observed for the 3 hormones we analyzed (indoleacetic acid, abscisic acid and salicylic acid) and a significant reduction in putrescine concentration occurred at 24h. A schematic representation of the different metabolite levels allows to visualize some metabolic pathways targeted by the JA treatment (S4 Fig).

In comparison, we observed that less metabolites are significantly altered by JA treatment in *oscoi1ab* plants (27 metabolites at 24h) and *oscoi2* plants (19 metabolites at 24h). These data pointed to specific metabolites targeted by JA with some being dependent on *oscoi2*, for instance in the AA category. We observed significant accumulation of quinic and shikimic acids in WT and *oscoi2* plants, while expression of the genes involved in the shikimate biosynthesis was dependent on *oscoi-2* (Fig 5c). However, while significative, the accumulation of these 2 metabolites is lower in *oscoi2* plants (S9 Table).

### Differential accumulation of phytoalexins in *oscoi* lines submitted to JA treatment

**Fig 8:**
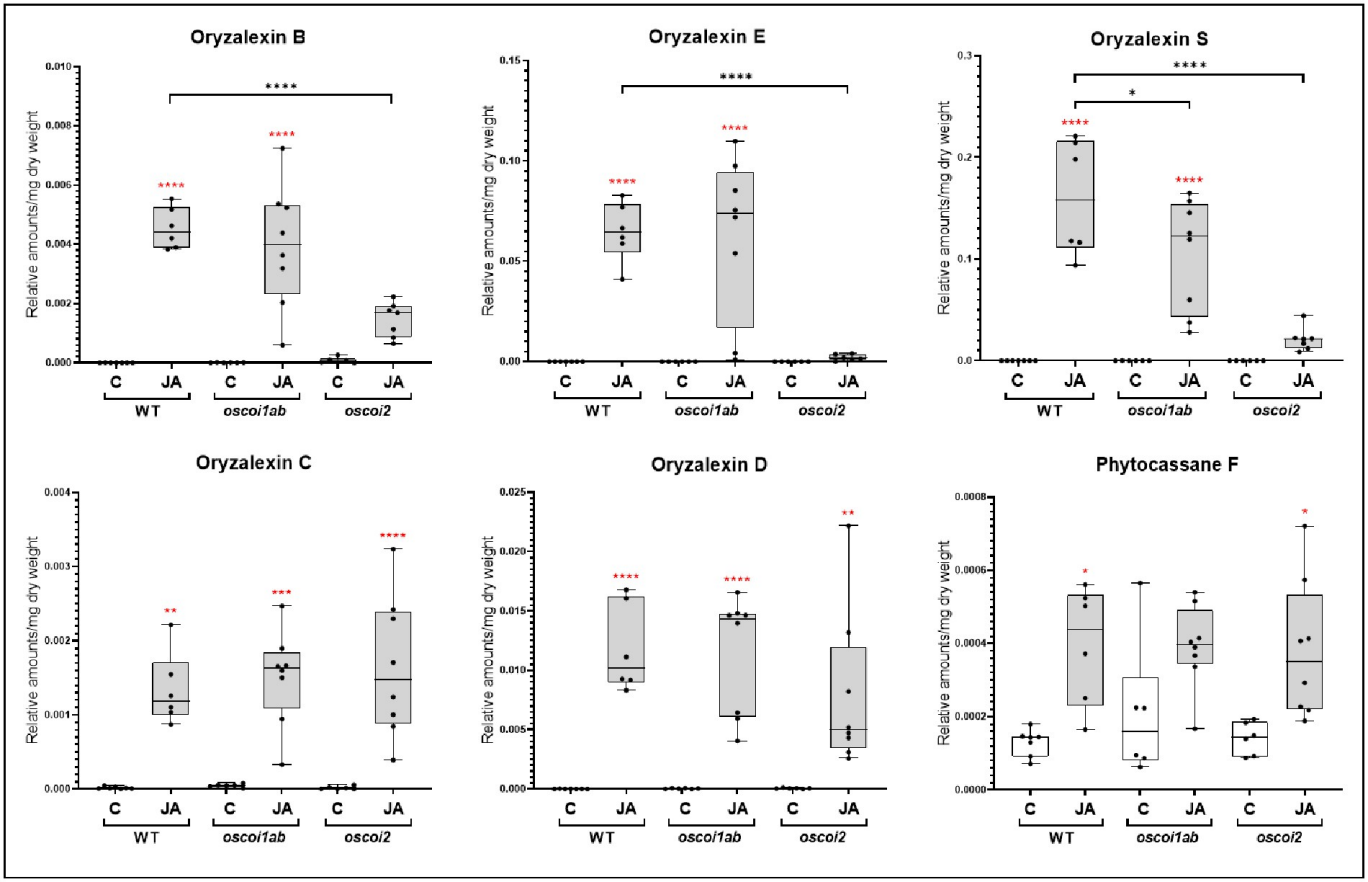
Phytoalexins accumulation in rice roots submitted to JA treatment. Phytoalexin levels in rice roots from control plants (C) and from plants submitted to a 24h JA treatment (5 μM). In the boxplots, whiskers denote minimum/maximum values, the box defines the interquartile range and the center line represents the median. Asterisks above the boxplots indicate significant differences between treated and control plants. Asterisks above the brackets indicate significant differences between lines and WT plants (One way-ANOVA with Tuckey’s multiple comparisons test, * p < 0.05, ** p < 0.01, *** p < 0.001, **** p < 0.0001).

The accumulation of 6 phytoalexins (Phytocassane F, Oryzalexins B, C, D, E and S) were measured in rice root systems treated by JA during 6 h and 24 h (Fig 8 and S5 Fig). In the mock-treated plants, no significant differences were detected between the amounts of phytoalexins in WT plants and *oscoi* lines. After 6h of JA treatment, when the DP biosynthesis genes are clearly induced in WT and *oscoi1ab* plants, we only observed a slight accumulation of Oryzalexins B and S in WT plants (~8 fold compared to control plants). After 24h, all the studied phytoalexins were accumulated in response to JA treatment in WT plants, at low level for phytocassane F (~3 fold) to high levels for phytoalexins B, E, S and D (>700 fold). We observed similar levels in *oscoi1ab* plants except for oryzalexin S which was slightly less induced, than in WT plants. We observed significantly less accumulation of Oryzalexins S, B and E in *oscoi2* plants compared to WT plants. Altogether, these data indicate a differential regulation for these phytoalexins, in an *OsCOI2*-dependent manner.

## Discussion

In this study, we explored the JA molecular responses in rice roots, trying to elucidate the specific roles of the JA rice receptors and specifically those of OsCOI2. We deciphered the molecular pathways controlled by OsCOI1a, OsCOI1b and OsCOI2 receptors through transcriptomic and metabolomic analyses and revealed the major role of OsCOI2 in the control of the JA response in roots.

### OsCOI2 is the main actor of the large transcriptional reprogramming induced by JA

Until recently, few publications focused on OsCOI2 function in rice plants. As OsCOI2 was not able to complement the *coi1* mutant in Arabidopsis [15], it was neglected and a key role for OsCOI2 in JA signaling was never suggested nor predicted. This study and two recent studies [20–21] converge to demonstrate OsCOI2 as the main actor of the massive transcriptional reprogramming observed after a JA challenge or a biotic stress in rice plants. Hence, a key role for OsCOI2 and only a partial involvement of OsCOI1a and OsCOI1b were described in the response of rice leaves to MeJA treatment [20]. In leaves attacked by the BPH insect, the major transcriptomic changes are controlled by OsCOI2, even if OsCOI1a/b and OsCOI2 can mediate shared downstream defenses [21]. In rice roots submitted to JA treatment, we did not identify any specific OsCOI1-dependent pathway. We observed some functional redundancy where rice genes were detected as DEGs both in *oscoi1ab* and *oscoi2* plants, however with usually a mitigated response in *oscoi2* plants. Expression patterns of *OsJAZs* and of a series of TFs including *OsMYC2* and *JA-MYB* perfectly illustrate such differential response levels. Taken together, these data validate the role of OsCOI2 as the main actor of transcriptional reprogramming in response to JA.

### A major role for *OsCOI2* in root growth inhibition by jasmonate

Root growth inhibition in response to JA treatment has been extensively described in plants [24]. In rice, we measured a ~60% root growth reduction for WT and *oscoi1ab* plants upon JA treatment and a ~30% growth reduction for *oscoi2* plants. In this study, we identified 32 down-regulated genes associated to cell-wall function in WT plants upon JA treatment from which 21 have an OsCOI2-dependent expression. These data indicate that in response to JA, expression patterns of genes involved in cell wall remodeling and expansion are correlated to the observed phenotypes. These data illustrate again the quantitative aspects of the differential expression levels between *oscoi1ab* and *oscoi2* plants.

The JA signaling pathways are subject to many layers of regulation. Under the control of OsCOI receptors and after degradation of OsJAZ repressors, specific TFs may be involved in the regulation of cell-growth related genes. In Arabidopsis, during JA-induced root growth inhibition, TFs from subclade bHLHIIIe (MYC2, 3 and 4) mediate the inhibition of primary root growth by JA, while TFs from subclade bHLH-IIId have repressive effects on JA signaling [24]. In our data, we identified several OsCOI2 dependent TFs, either up- or down-regulated, that are known to be involved in root development (S7 Table). They represent good candidates for controlling expression of cell-wall related genes.

### OsCOI2 is involved in the control of the phenylpropanoid pathway

Phenylpropanoid metabolism contributes to both plant development and plant-environment interactions [25]. Phenylalanine, the key precursor of the PPP at the interface between the primary metabolites and the secondary metabolites, derives from the shikimate pathway [26]. We showed that genes involved in shikimate and phenylalanine synthesis were induced by JA in an OsCOI2-dependent manner. At the metabolite level, we detected a strong accumulation of quininic and shikimic acids at 6h and 24h after JA treatment, as well as a moderate accumulation of phenylalanine. These metabolites were induced in all the genotypes although their accumulation was lower in *oscoi2* plants.

Many secondary metabolites are produced in the PPP including two major branches leading to lignins and flavonoids production. Transcriptional regulation plays a central role in the regulation of their biosynthesis and it can be modulated by diverse signaling pathway, including phytohormones related pathways [25]. For instance, on the 85 flavonoid biosynthesis genes identified in rice, 23 are known to be induced by JA [27]. In rice roots, we identified 10 PPP genes as DEGs in WT plants and the expression of 7 of them was OsCOI2-dependent. Numerous TFs have been identified as regulators of the PPP biosynthesis genes, especially from the MYB and bHLH families [25, 28]. In rice, OsMYB30 was identified as a master PPP regulator. It can activate the *Os4CL3* and *Os4CL5* genes to induce lignin accumulation and enhance *Magnaporthae oryzae* resistance [29]. In rice roots, we observed that *OsMYB30*, *Os4CL3* and *OsC4CL5* are induced by JA in an OsCOI2-dependent manner. It would be interesting to perform lignin quantification in both plants to determine if lignification in response to JA is *oscoi2*-dependent. In another study, OsMYB30 was shown to directly bind to the promoters of *OsPAL6* and *OsPAL8* and regulate their expression in response to the BPH insect attack, resulting in accumulation of salicylic acid and lignin and enhanced resistance [30]. *OsPAL6* and *OsPAL8* were not induced in our data, as well as other insect-related genes such as *OsLIS* or *OsCAS*, indicating that the JA signal was not sufficient to induce antiherbivore-defense in roots. In addition, OsMYB30 and the TFs OsMYB55 and OsMYB110 were shown to activate shikimate and cinnamate/monolignol pathways and enhance resistance to both fungal and bacterial pathogens [31]. In our study, both *OsMYB55* and *OsMYB110* were induced by JA in an OsCOI2-dependent manner. All these data indicate that OsCOI2 is a master regulator of phenylpropanoid biosynthesis genes.

### OsCOI2 controls diterpene phytoalexins biosynthesis

The biosynthesis of rice phytoalexins can be regulated by various biotic and abiotic stress and phytohormones such as JA [32, 33]. In rice, three biosynthesis gene clusters are mainly responsible for DP biosynthesis [34, 35]. Some biosynthesis genes such as *OsKSL8* and *OsKSL10*, however, are located outside these clusters. Majority of these genes can be induced by MeJA in rice leaves [35]. In this study, we showed that JA also induced their expression in roots. Importantly, their induction is OsCOI2 dependent, as it was already shown in rice leaves [20].

JA-responsive TFs are key players known to coordinate the regulation of secondary metabolism pathway [36]. In rice, four transcriptional activator of DP biosynthesis genes have been characterized. OsTGAP1, a bZIP TF specifically expressed in rice roots, mainly regulates expression of momilatone biosynthesis genes and in a lesser extent expression of phytocassanes biosynthesis genes [37, 38]. *OsDPF*, a bHLH TF inducible in leaves by blast infection, copper chloride, UV and JA [39] and in roots by *Pythium arrhenomanes* infection [40], regulates expression of some DP biosynthesis genes, notably *OsCPS2* and *OsCYP99A2*. OsbHLH026, another bHLH TF, enhances the disease resistance of rice to *Xanthomonas oryzae* through the activation of *OsCPS2* and *OsKSL6* [41]. Recently, OsWRKY10 was identified as a master regulator of DP biosynthesis acting directly on several DP biosynthesis genes [42]. In our data, only *OsDPF* displays an OsCOI2-dependent expression upon JA treatment suggesting that it is the key regulator of DP biosynthesis genes in rice roots. *OsWRKY10* is induced by JA in *oscoi2* plants but at lower level than in WT plants. Such induction level in *oscoi2* plants might be not sufficient to induce the DP biosynthesis genes or it may require coregulator(s) not expressed in these conditions or *oscoi2*-dependent.

If transcriptomic analyses clearly showed that the expression of DP biosynthesis genes is OsCOI2-dependent, metabolite analyses of phytoalexin levels gave contrasting results. Oryzalexins S, B and E accumulation upon JA treatment is OsCOI2-dependent while Phytocassane F and Oryzalexins C and D display the same accumulation patterns whatever the genotype. Differential accumulation of Oryzalexins is difficult to explain as they result from the same biosynthesis pathways (except Oryzalexin S) and all the known genes involved in Oryzalexins biosynthesis appears to be OsCOI2-dependent. Other genes involved in oryzalexins biosynthesis, such as specific CYPs, which are not well identified could possibly differentially participate to their biosynthesis. Alternatively, the lower activation of *OsWKRY10* in *oscoi2* plants, can be sufficient to induce phytoalexin biosynthesis, even if *OsDPF* is not expressed.

Collectively, these data reveal that *OsCOI2* regulates DP biosynthesis and that its function is not redundant with that of *OsCOI1a* and *OsCOI1b* JA receptors. Due to the importance of DP in plant resistance to pathogens, the role of OsCOI2 as a master regulator of plant defense should be explore. Enhanced susceptibility to the rice insect PBH has been established in leaves from *oscoi2* plants [21]. In roots, it was shown that DP are important players in basal and induced resistance against the rice nematode *Meloidogyne graminicola* [43]. Testing the level of susceptibility to *M*. *graminicola* in the *oscoi2* plants would give important response on the signaling pathway involved in rice defenses.

### Primary metabolism shifts to secondary metabolism under the control of OsCOI2

In response to stress, a metabolic shift occurs in plant cells to produce protective compounds, especially specialized secondary metabolites. For instance, amino acids are precursors of a number of secondary metabolites and function as a bridge between primary and secondary metabolism [44]. The metabolic shift must be coordinated to support the resource demands of secondary metabolites production without suppressing the primary metabolism and the production of growth-stimulating metabolites [45]. JA acts as a major regulatory hub mediating the shift from the central primary metabolism to the secondary metabolism and allowing the production of defense compounds in response to stress [44–47]. Hence, JA regulates the trade-off between plant growth and defense responses, illustrating the trade-off growth-defense concept [48].

The trade-off process cannot be described as a simple redistribution of resources between growth and protection but rather is an active plant strategy requiring a large reprogramming of gene expression [44, 45, 47]. In our data, we observed such large transcriptional reprograming in rice roots in response to JA treatment. Photosynthesis related genes as well as cell-growth related genes were massively down-regulated by JA, while numerous secondary metabolites biosynthesis genes were up-regulated. At the metabolic level, we also detected large modifications in the primary metabolite contents. Most of the AA as well as TCA cycle acids showed decreased levels in response to JA. Conversely, components of the shikimate pathway and phenylalanine, the PPP precursors, display high levels of accumulation. Levels of secondary metabolites were tested through the measurement of oryzalexins and phytocassanes contents. We showed that a 6h JA treatment was not sufficient to detect a synthesis of these metabolites whereas biosynthesis genes were up-regulated at this time. However, we could observe a massive accumulation of these phytoalexins after 24h of treatment.

In this study, we demonstrate that in rice roots the metabolic shift observed in response to JA is mainly OsCOI2-dependent. This dependency is clearly shown at the molecular level where several inducible pathways are completely abolished in *oscoi2* plants. Overall, our data suggest that OsCOI2 controls the growth-defense trade-off in response to JA.

### How OsCOI2 can regulate specific signaling pathways?

Activation of specific OsCOI2-dependent signaling pathways can be trigger by specific TF. In our data, we identified more than 150 OsCOI2-dependent TFs representing good candidates for the regulation of the OsCOI2-specific pathways. Upstream of the TFs, JAZs repressors ensure the link between JA perception by COI receptors and TF de-repression. In response to JA, overexpression of almost all *OsJAZ* still occur in *oscoi2* mutant, even weakened, while expression of *OsJAZ-2*, *-9* and *-13* is fully OsCOI2-dependent. Interactions between OsJAZ repressors and OsCOI2 have been already identified but recent data with contradictory results show how difficult it is to assume specific interactions between OsCOI2 and OsJAZs. In presence of JA-Isoleucine, OsCOI2 is able to interact with all OsJAZs—except OsJAZ14—in Okumura *et al* [49] whereas it only interacts with OsJAZ-3, -4, -6, -7, -10 and -15 in Wang *et al* [21]. *In silico* docking simulation indicates that OsCOI2 has a larger ligand-binding pocket than OsCOI1 proteins suggesting that JA-Ile or larger ligands can take flexible conformation appropriate for the binding to the OsJAZs of variable conformations [49]. OsCOI2-OsJAZ interaction could be more efficient and could explain the high number of JA responses OsCOI2-dependent. These hypotheses remain to be explored to identify the molecular mechanisms involved in JA perception and signaling in rice root.

## Materials & methods

### Edited rice lines

T3 homozygous lines in Kitaake background were used in this study. The *oscoi1a-1/b-1* line is edited both in the *OsCOI1a* (4 nt deletion) and *OsCOI1b* genes (A deletion) and the *oscoi2-2* line is edited in the *OsCOI2* gene (T deletion and -36 nt deletion) as previously described in Nguyen et al. [22]. They are named *oscoi1ab* and *oscoi2* in the text.

### Whole genome sequencing and potential off-targets analysis

Young leaves from WT (Kitaake), *oscoi1ab* and *oscoi2* rice plants were used to extract genomic DNA for SQK-LSK109 libraries preparation as described in Serret *et al* [50]. Genome sequencing was performed on a Nanopore MinION Flow Cell R9.4.1 (Oxford technologies, Oxford Science Park, UK). The long-read sequences were assembled and evaluate as described in Adam *et al* [51]. Sequences of each chromosome were aligned and plotted using the minimap2 aligner implemented in the D-GENIES tool (https://dgenies.toulouse.inra.fr/run, [52]). Potential off-target sites were searched in the Kitaake sequence with the Primer3.2.3.7 tool included in the Geneious Prime^®^ 2023.2.1 package, using the full 20-bp target sequence plus the NGG PAM sequence as query and a maximum mismatch of 5. Putative off-target mutations (SNP/indels) were searched in the *oscoi1ab* and *oscoi2* sequences at the potential off-target sites.

### Hormone treatments

For RNA-seq experiments, three samples were selected from the five independent biological samples performed in Nguyen *et al* [22]. Briefly, crown root tips (around 1 cm) from 20–24 plants were collected 6 h after JA treatment (5 μM) or mock treatment (DMSO) and immediately frozen in liquid nitrogen. For metabolomics analyses, root systems from 16–20 plants were collected 6 h and 24 h after JA treatment (5 μM). Nine independent biological samples were collected and immediately frozen in liquid nitrogen.

### RNA-seq libraries, sequencing and mapping

The RNA-seq analysis was made on three biological replicates per sample. cDNA libraries preparation and sequencing were performed by Novogene (Novogene, Cambridge, UK) using the Illumina NovaSeq platform generating 150-bp paired-end reads. The quality of each library was controlled using FastQC. Reads were trimmed with CutAdapt using the following criteria: the first 12 bases of all reads were eliminated, contaminating adapters were removed, low quality bases (Q<30) were trimmed out from both the 5- and 3-ends of reads, and reads less than 35 nucleotides were discarded. Clean reads were mapped against the *Oryza sativa* reference genomes (MSU7 and RAP-DB annotations) using HiSat2. Mapped reads were assembled into transcripts with StringTie using a reference annotation. Count files were generated from GTF files using an in-house script and merged to obtain files including the annotated genes from both annotations.

### Gene expression analyses

DEGs were detected using EdgeR [53] running in the DIANE (Dashboard for the Inference and Analysis of Networks from Expression data) shinny application [54]. This analysis allowed to examine the expression of 30.898 rice genes from which selection criteria for DEGs were defined as log_2_Fold Change (log_2_FC) ≥1 or ≤−1, False Discovery Rate (FDR) <0.01 and a minimal gene count sum across libraries of 180. Gene Ontology Enrichment analyses were performed on the Rice Genome Hub website (https://rice-genome-hub.southgreen.fr) using a p-value cutoff of 0.01. The MapMan tool (https://mapman.gabipd.org/home, [55]) was used to visualize the various metabolic pathways related to DEGs. The heatmaps were constructed with the Morpheus software (https://software.broadinstitute.org/morpheus) using log_2_FC values of the DEGs. The Venn diagrams were performed with the Venny2.1 tool (https://bioinfogp.cnb.csic.es/tools/venny). Gene expression data were validated by RT-qPCR performed on 5 biological replicates (the three biological replicates used for RNA-seq plus two independent biological replicates) as previously described [22]. Primers used for amplification are listed in S10 Table.

### Metabolite extraction

Total metabolite extraction was executed following the protocol described earlier by Gemmer *et al* [56], albeit with few modifications. In brief, the rice root samples were pulverized using a Retsch-ball mill (MM 400, Retsch, Germany) for 1.5 min at 20 Hz. The homogenized root samples were lyophilized and then reconstituted in 900 μl of solvent mixture of methanol, chloroform and water (3:2:4, v/v), enriched with 8 μg/ml of ^13^C-sorbitol, 13.2 μg/ml of sinigrin and 4.5μg/ml of methyl nonadecanoate (Sigma-Aldrich, USA) to serve as internal quantitative standards. The mixture was shaken for half a minute with intermittent cooling on ice to mitigate any heat generation and degradation of metabolites. The mixture was then centrifuged for 14,000 X g for 3 min at 4°C. The upper polar phase was collected and subjected to filtration using a 0.22 μm PVDF syringe filter (Carl Roth GmbH, Germany). These refined extracts were then subjected for comprehensive metabolite profiling and were preserved at -80°C pending analysis via Gas Chromatography—Mass Spectrometry (GC-MS). To augment data integrity and precision, quality controls (QC) comprising 40 μl aliquots from each sample amalgamated were incorporated in every analytical batch constituting one-fifth of the total samples. This strategy was instrumental in enhancing data quality and expunging anomalies from the final data.

### Metabolite profiling by GC-MS

Post-extraction, 10 μl aliquot of the filtered extract from the samples was subjected to desiccation in a vacuum concentrator (Christ, Germany) for a duration of 45 min, devoid of any heating. The samples were then prepared for online derivatization using the Multi-Purpose Sampler (MPS, Gerstel, Germany) PAL system (Chromtech evolution). A volume of 30 μl of methoxamine hydrochloride (20 mg/ml in pyridine) was introduced to these samples. The samples were then shaken for 30 min at a temperature of 45°C. Subsequently, 45μl of BSTFA (N,O-Bis(Trimethylsilyl)trifluoroacetamide) and 5 μl alkane-standard (C10-C28; 6 mg/ml—Retention time standard) were added and the samples were shaken additionally for 120 min at 45°C.

All the samples along with 20% of quality controls, were analysed with GC-MS (GC-qTOF system -7890B/7200, Agilent, Santa Clara, USA). One μl of these derivatized samples was injected at 250°C in both splitless mode and a 20:1 split mode, with a helium gas flow set to 1 ml min^-1^. Chromatographic separation was achieved using an HP5-MS GC-Column (Agilent, 30 m, 0.25 mm, 0.25 μm). The temperature program commenced at 60°C, followed by a linear ramp of 10°C/min to 320°C, where it was maintained for 3 min. The EI source was set to 70eV, and the temperatures for the transfer line, source, and quadrupole were set to 290°C, 230°C and 150°C, respectively.

The raw data was processed using MassHunter Qualitative Analysis software (Agilent, B.10.00) and MassHunter Quantitative Analysis software for QTOF (Agilent, B.08.00). The mass spectra library NIST 14 (National Institute of Standards and Technology) and our in-house library were utilized for the identification and confirmation of the chromatographic peaks. Peak areas were initially normalized for intra- and inter batch corrections using the web-based Galaxy tool [57] and subsequently with the respective internal standard and the respective sample dry weight. Extreme outliers, defined as values 3-fold higher than the box plot whiskers, were excluded.

### Diterpenoid metabolites

After phase separation during the above-mentioned metabolite extraction procedure, the lower non-polar chloroform phase of the samples was subjected to filtration using a 0.22 μm PTFE syringe filter (Carl Roth GmbH, Germany). The samples were then prepared for online derivatization using the Multi-Purpose Sampler (MPS, Gerstel, Germany) PAL system (Chromtech evolution). Subsequently, 45μl of BSTFA (N,O-Bis(Trimethylsilyl)trifluoroacetamide) was added and the samples were shaken for 120 min at 45°C. All samples were analyzed by GC-TOF-MS (7890B/7200, Agilent, Santa Clara, CA, USA). One μl of the filtered samples was injected at 250°C in splitless mode with a helium gas flow set to 1 mL/min. Chromatographic separation was achieved using a HP5-MS GC-Column (Agilent, 30 m, 0.25 mm, 0.25 μm). The temperature program commenced at 60°C, followed by a linear ramp of 10°C/min to 325°C, where it was maintained for 3 min. Throughout the run, the temperature for the transfer line, source, and the quadrupole were set to 290°C, 230°C, and 150°C, respectively. The ionization was performed with chemical ionization (CI) and recorded in scan mode. The raw data was processed using MassHunter Qualitative Analysis software (Agilent, B.10.00) and MassHunter Quantitative Analysis software for QTOF (Agilent, B.08.00). Specific molecular ions obtained due to the application of QTOF (which is a high-resolution MS), led to the identification and confirmation of the chromatographic peaks for multiple diterpene compounds. Pseudo-molecular ions and the retention times of the various diterpenes analysed are as follows: Phytocassane F (333.2053, 15.887 min), Oryzalexins S (269.2276, 22.194 min), B (375.2684, 20.554 min), C (301.2047, 22.738 min), D (269.2251, 22.128) and E (269.2267, 21.621 min). Peak areas were initially normalized for intra- and inter batch corrections using the web-based Galaxy tool [57] and subsequently with the internal standard (methyl nonadecanoate) and the respective sample dry weight.

### Statistical analyses

Metabolomic data were analyzed using GraphPad Prism (version 9.3.0). Values were considered statistically significant when p < 0.05.

## Supporting information

S1 Fig Whole genome sequences alignment. Genomic sequences from *oscoi1ab* and *oscoi2* plants were aligned to the Kitaake genomic sequence used in this study.
(DOCX)

S2 Fig Principal component analysis of RNA-seq data originated from the 18 libraries used in this study. (DOCX)

S3 Fig Validation of RNAseq data by RT-qPCR. a. RNAseq expression data from 11 rice genes in response to JA treatment in WT, oscoi1ab and oscoi2 plants. Asterisks indicate Differentially Expressed Genes as determined by EdgeR (log2FC ≥1 or ≤-1 and FDR ≤0.01). b. RT-qPCR values obtained from the same genes. Bars represent the mean of log2(relativeFC) ± SE from five biological replicates. Asterisks indicate significant differences between control and JA treated plants (n = 5, t-test, p-value ≤0.01).
(DOCX)

S4 Fig Primary metabolism of rice roots submitted to JA treatment. The key color represents the Log_2_FC values between JA-treated samples (5 μM, 24h) and controls samples from WT and *oscoi* plants.
(DOCX)

S5 Fig Phytoalexins accumulation in rice roots submitted to JA treatment. Phytoalexin levels in rice roots from control plants (C) and from plants submitted to a 6h JA treatment (5 μM). In the boxplots, whiskers denote minimum/maximum values, the box defines the interquartile range and the center line represents the median. Asterisks above the boxplots indicate significant differences between treated and control plants. Asterisks above the brackets indicate significant differences between WT plants and *oscoi* lines (One way-ANOVA with Tuckey’s multiple comparisons test, * p < 0.05, ** p < 0.01, *** p < 0.001, **** p < 0.0001).
(DOCX)

S1 Table Whole genome sequencing and assembly statistics. (XLSX)

S2 Table Potential off-targets sites and mutations detected in the *oscoi* mutant lines. (XLSX)

S3 Table RNA-seq libraries used in this study. (XLSX)

S4 Table Lists of DEGs identified in *oscoi* mutant lines versus WT plants, in absence of any treatment. (XLSX)

S5 Table Lists of DEGs identified in JA treated plants versus control plants in WT, *oscoi1ab* and *oscoi2* plants. (XLSX)

S6 Table Lists of genes and expression data used to establish the heatmaps shown in this study. (XLSX)

S7 Table Lists of DEGs encoding transcription factors. (XLSX)

S8 Table GO enrichment of the DEGs identified in this study. (XLSX)

S9 Table Metabolite levels in rice roots submitted to JA treatment. (XLSX)

S10 Table List of primers used for RT-qPCR. (XLSX)
