# Supplementary material for: Transcriptomic and metabolomic reveal OsCOI2 as the jasmonate-receptor master switch in rice root
Source: PLoS One. 2024 Oct 28;19(10):e0311136. doi: 10.1371/journal.pone.0311136 (PMC11516173; doi:10.1371/journal.pone.0311136)
Supplement: S1 Fig — Genomic sequences from oscoi1ab and oscoi2 plants were aligned to the Kitaake genomic sequence used in this study. (DOCX) [file pone.0311136.s001.docx]

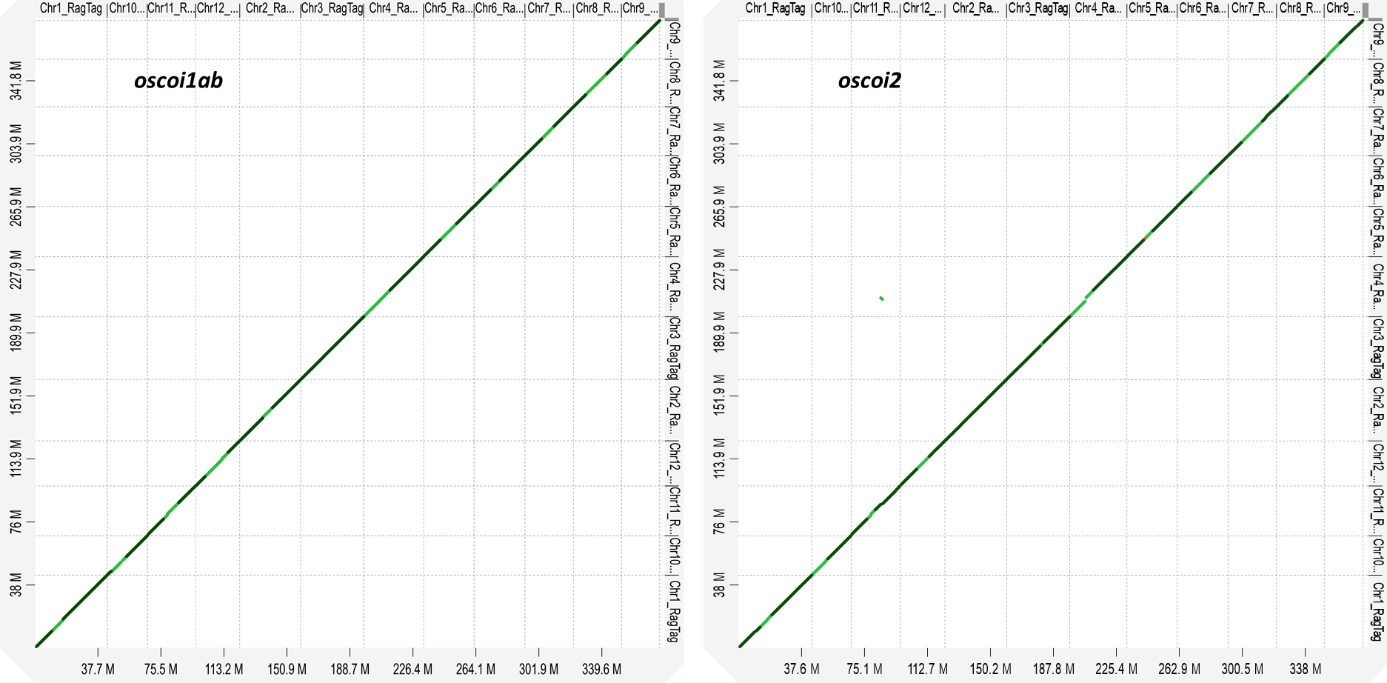


**S1 Fig.** Whole genome sequences alignment. Genomic sequences from *oscoi1ab* and *oscoi2* plants were aligned to the Kitaake genomic sequence used in this study.
