# Supplementary material for: Transcriptomic and metabolomic reveal OsCOI2 as the jasmonate-receptor master switch in rice root
Source: PLoS One. 2024 Oct 28;19(10):e0311136. doi: 10.1371/journal.pone.0311136 (PMC11516173; doi:10.1371/journal.pone.0311136)
Supplement: S2 Fig — (DOCX) [file pone.0311136.s002.docx]

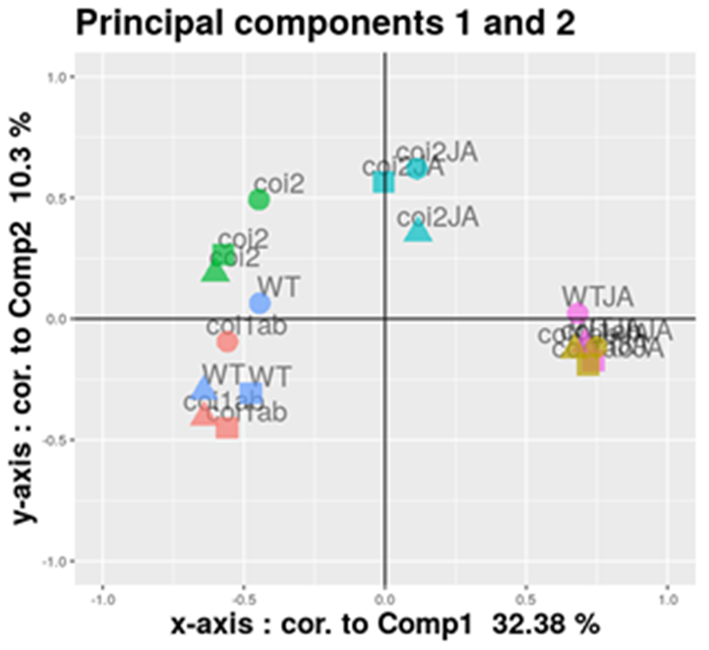


**S2 Fig.** Principal component analysis of RNA-seq data originated from the 18 libraries used in this study.
