# Supplementary material for: Transcriptomic and metabolomic reveal OsCOI2 as the jasmonate-receptor master switch in rice root
Source: PLoS One. 2024 Oct 28;19(10):e0311136. doi: 10.1371/journal.pone.0311136 (PMC11516173; doi:10.1371/journal.pone.0311136)
Supplement: S3 Fig — a. RNAseq expression data from 11 rice genes in response to JA treatment in WT, oscoi1ab and oscoi2 plants. Asterisks indicate Differentially Expressed Genes as determined by EdgeR (log2FC ≥1 or ≤-1 and FDR ≤0.01). b. RT-qPCR values obtained from the same genes. Bars represent the mean of log2(relativeFC) ± SE from five biological replicates. Asterisks indicate significant differences between control and JA treated plants (n = 5, t-test, p-value ≤0.01). (DOCX) [file pone.0311136.s003.docx]

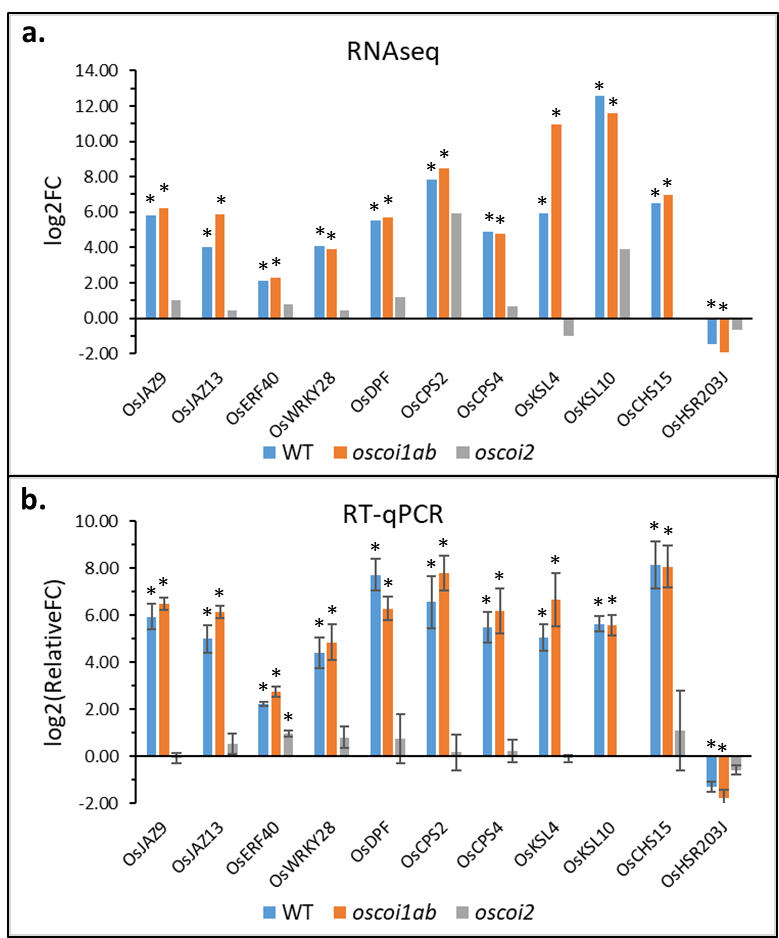


**S3 Fig.** **Validation of RNAseq data by RT-qPCR**. **a.** RNAseq expression data from 11 rice genes in response to JA treatment in WT, *oscoi1ab* and *oscoi2* plants. Asterisks indicate Differentially Expressed Genes as determined by EdgeR (log_2_FC ≥1 or ≤-1 and FDR ≤0.01). **b.** RT-qPCR values obtained from the same genes. Bars represent the mean of log_2_(relativeFC) ± SE from five biological replicates. Asterisks indicate significant differences between control and JA treated plants (n=5, t-test, p-value ≤0.01).
