# Supplementary material for: Transcriptomic and metabolomic reveal OsCOI2 as the jasmonate-receptor master switch in rice root
Source: PLoS One. 2024 Oct 28;19(10):e0311136. doi: 10.1371/journal.pone.0311136 (PMC11516173; doi:10.1371/journal.pone.0311136)
Supplement: S4 Fig — The key color represents the Log2FC values between JA-treated samples (5 μM, 24h) and controls samples from WT and oscoi plants. (DOCX) [file pone.0311136.s004.docx]

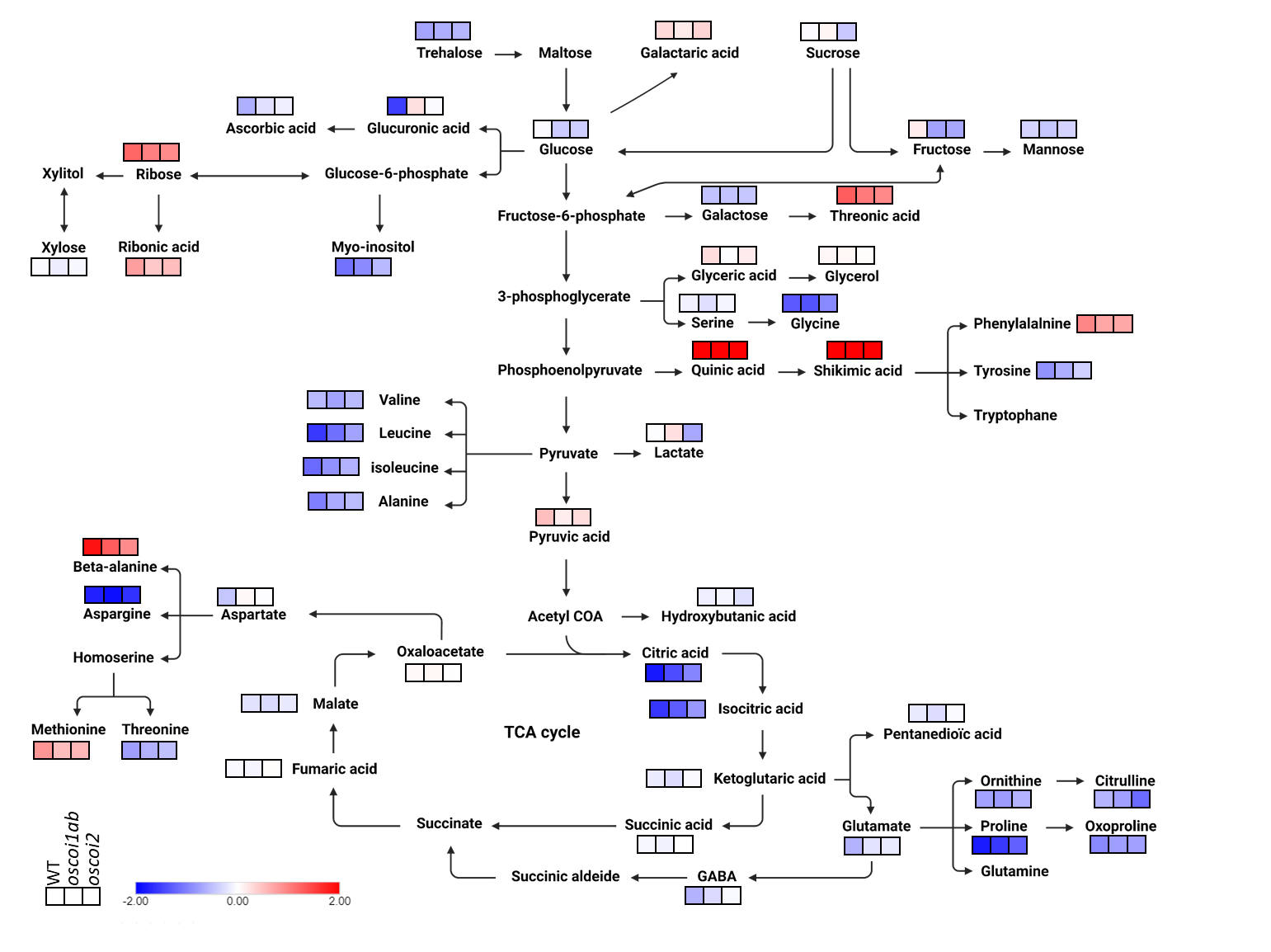


**S4 Fig.** Primary metabolism of rice roots submitted to JA treatment. The key color represents the Log_2_FC values between JA-treated samples (5 µM, 24h) and controls samples from WT and *oscoi* plants.
