# Supplementary material for: Transcriptomic and metabolomic reveal OsCOI2 as the jasmonate-receptor master switch in rice root
Source: PLoS One. 2024 Oct 28;19(10):e0311136. doi: 10.1371/journal.pone.0311136 (PMC11516173; doi:10.1371/journal.pone.0311136)
Supplement: S5 Fig — Phytoalexin levels in rice roots from control plants (C) and from plants submitted to a 6h JA treatment (5 μM). In the boxplots, whiskers denote minimum/maximum values, the box defines the interquartile range and the center line represents the median. Asterisks above the boxplots indicate significant differences between treated and control plants. Asterisks above the brackets indicate significant differences between WT plants and oscoi lines (One way-ANOVA with Tuckey’s multiple comparisons test, * p < 0.05, ** p < 0.01, *** p < 0.001, **** p < 0.0001). (DOCX) [file pone.0311136.s005.docx]

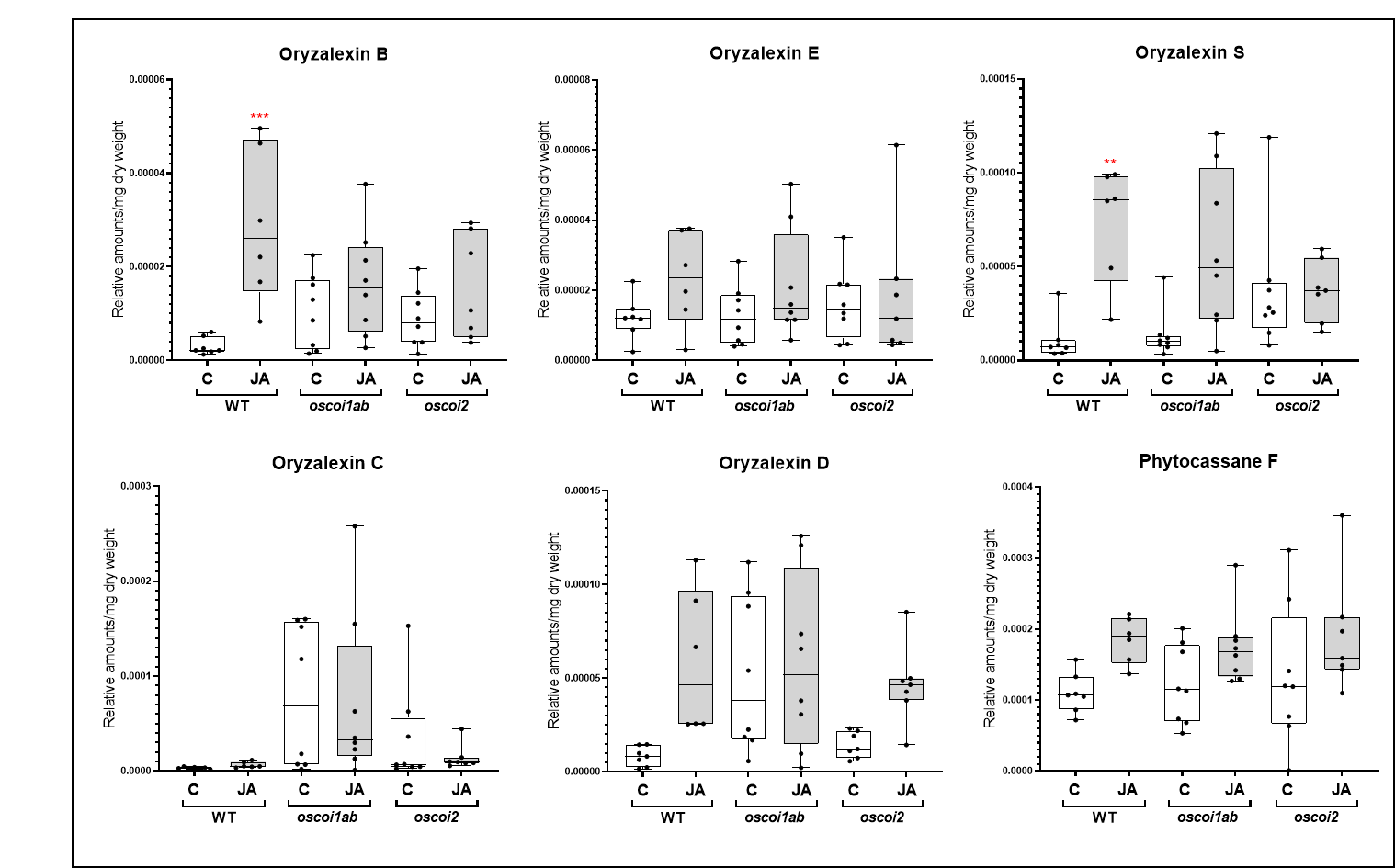


**S5 Fig.** Phytoalexins accumulation in rice roots submitted to JA treatment. Phytoalexin levels in rice roots from control plants (C) and from plants submitted to a 6h JA treatment (5 µM). In the boxplots, whiskers denote minimum/maximum values, the box defines the interquartile range and the center line represents the median. Asterisks above the boxplots indicate significant differences between treated and control plants. Asterisks above the brackets indicate significant differences between lines and WT plants (One way-ANOVA with Tuckey’s multiple comparisons test, * p < 0.05, ** p < 0.01, *** p < 0.001, **** p < 0.0001).
